# Sepsis-induced selective parvalbumin interneuron phenotype loss and cognitive impairments may be mediated by NADPH oxidase 2 activation in mice

**DOI:** 10.1186/s12974-015-0401-x

**Published:** 2015-09-29

**Authors:** Mu-Huo Ji, Li-Li Qiu, Hui Tang, Ling-Sha Ju, Xiao-Ru Sun, Hui Zhang, Min Jia, Zhi-Yi Zuo, Jin-Chun Shen, Jian-Jun Yang

**Affiliations:** Department of Anesthesiology, Jinling Hospital, School of Medicine, Nanjing University, 305 East Zhongshan Road, Nanjing, 210002 China; Department of Anesthesiology, University of Virginia Health System, Charlottesville, VA USA

**Keywords:** Parvalbumin interneurons, Cognition, Sepsis, NADPH oxidase, Oxidative stress

## Abstract

**Background:**

Sepsis-associated encephalopathy (SAE) is a diffuse brain dysfunction caused by many pathological events, including neuroinflammation and oxidative stress damage. Increasing evidence suggests that parvalbumin (PV) interneurons play a key role in the cognitive process, whereas the dysfunction of these interneurons has been implicated in a number of major psychiatric disorders. Here, we aimed to investigate whether enhanced inflammation and oxidative stress-mediated PV interneuron phenotype loss plays a role in sepsis-induced cognitive impairments.

**Methods:**

Male C57BL/6 mice were subjected to cecal ligation and puncture or sham operation. For the interventional study, the animals were chronically treated with a nicotinamide adenine dinucleotide phosphate (NADPH) oxidase inhibitor, apocynin, at 5 mg/kg. The mice were euthanized at the indicated time points, and the brain tissues were harvested for determination of the PV, membrane subunit of NADPH oxidase gp91^phox^, and markers of oxidative stress (4-hydroxynonenal and malondialdehyde) and inflammation (tumor necrosis factor alpha (TNF-α), interleukin (IL)-1β, IL-6, and IL-10). A separate cohort of animals was used to evaluate the behavioral alterations by the open field and fear conditioning tests. Primary hippocampal neuronal cultures were used to investigate the mechanisms underlying the dysfunction of PV interneurons.

**Results:**

Sepsis resulted in cognitive impairments, which was accompanied by selective phenotype loss of PV interneurons and increased gp91^phox^, 4-hydroxynonenal, malondialdehyde, IL-1β, and IL-6 expressions. Notably, these abnormalities could be rescued by apocynin treatment.

**Conclusion:**

Selective phenotype loss of PV interneurons, as a result of NADPH oxidase 2 (Nox2) activation, might partly contribute to cognitive impairments in a mouse model of SAE.

## Background

Sepsis-associated encephalopathy (SAE) is a common brain dysfunction induced by systemic responses to infection, which often presents serious long-term cognitive impairments that can affect up to 70 % of patients with severe sepsis, leading to poor quality of life and increased mortality [1–5]. Several potential mechanisms, including oxidative stress, inflammation, neurotransmission disturbance, mitochondrial dysfunction, and cell death, have been proposed to be involved in the pathogenesis of SAE [5], but how these pathological factors lead to cognitive impairments remains largely to be elucidated.

Parvalbumin (PV) interneurons are a subset of inhibitory GABAergic neurons that control the excitability of post-synaptic pyramidal neurons [6]. These interneurons are critically involved in the generation of gamma oscillations, which facilitates the information processing during sensory perception and cognitive tasks [6–9]. By contrast, dysfunction or phenotype loss of PV interneurons (represented by decreased expression of GABA-related genes such as the 67-kDa isoform of glutamate decarboxylase or PV) has been implicated in cognitive deficits associated with a number of major psychiatric disorders, including schizophrenia [10], Alzheimer’s disease [11], and depression [12]. Although the precise mechanism underlying dysfunction of PV interneurons remains unknown, oxidative stress has been realized to be a contributing factor because of the high metabolic requirements of these interneurons [13–15]. In particular, superoxide overproduction as a result of nicotinamide adenine dinucleotide phosphate (NADPH) oxidase 2 (Nox2) activation leads to the reduced expression of GABAergic markers and consequent loss of inhibitory capacity of PV interneurons [16, 17]. Presently unknown, however, is whether Nox2 activation-induced PV interneuron dysfunction is involved in the sepsis-induced cognitive impairments.

Herein, we investigated whether the expression of PV in the prefrontal cortex (PFC) and hippocampus is altered after sepsis development and whether gp91^phox^ activation, the catalytic subunit of Nox2, contributes to these changes in a mouse model of SAE.

## Materials and methods

### Animals

Three hundred and sixty male C57BL/6 mice (3–4 months, 25–32 g) were purchased from the Animal Center of Jinling Hospital, Nanjing, China. The study protocol was approved by the Ethics Committee of Jinling Hospital, Nanjing University, and all procedures were performed in accordance with the Guideline for the Care and Use of Laboratory Animals from the National Institutes of Health, USA. The animals were housed under a 12-h light/dark cycle in a temperature-controlled room at 24 ± 1 °C with free access to food and water.

### Sepsis model

**Fig. 1:**
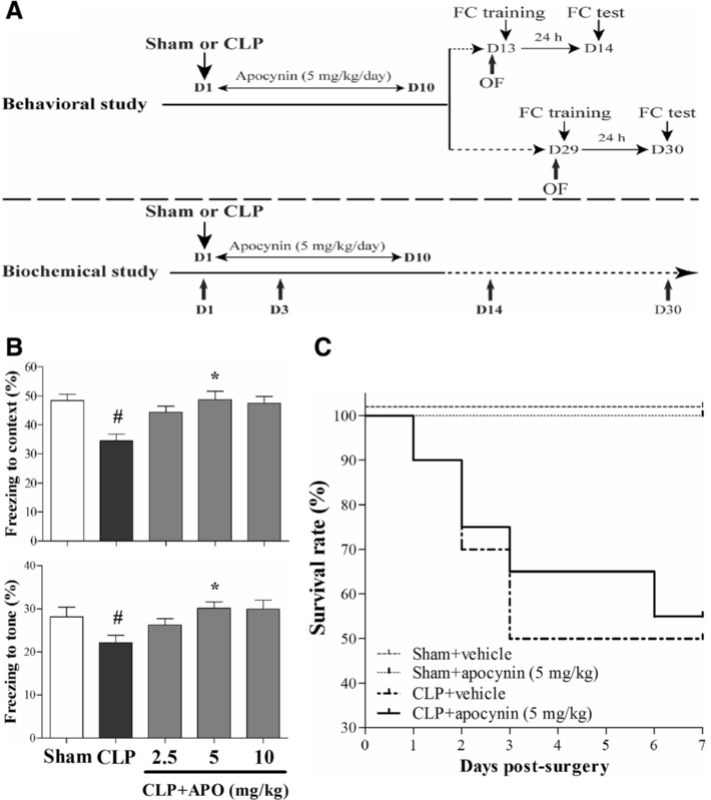
**a** Schematic timeline of the experimental procedures. **b** Dose-response of apocynin on the fear conditioning test in mice. Data are presented as mean ± SEM (*n* = 10–12). ^#^ *p* < 0.05 vs the sham group; *p < 0.05 vs the CLP group. **c** Apocynin treatment did not affect the survival rate of the sepsis mice (*n* = 12–20)

The mice were subjected to cecal ligation and puncture (CLP) as previously described [18]. Briefly, the mice were anesthetized with 2 % sodium pentobarbital in saline (50 mg/kg, intraperitoneally; Sigma, USA). A laparotomy was performed with a 1-cm midline incision through the linea alba. The cecum was isolated carefully and then ligated with 4.0 silk below the ileocecal junction, approximately 0.8 cm from the distal end. The cecum was then perforated twice with a sterile 22-gauge needle and was gently squeezed to extrude the fecal contents into the peritoneal cavity. The cecum was then returned to the peritoneal cavity, and the laparotomy was closed with 4.0 silk sutures. Sham-operated mice had the cecum isolated and then returned to the peritoneal cavity, without ligation or puncture of the cecum. All mice received subcutaneous normal saline resuscitation (20 ml/kg of body weight), and antibiotic therapy (ertapenem, 20 mg/kg; Merck Research Laboratory, USA) begun immediately after the surgery and once daily for a total of 3 days. The entire procedure was completed within 8 min. All animals were returned to their cages with free access to food and water. The flow chart for the study protocol was summarized in Fig. 1a.

### Apocynin treatment

Apocynin (Sigma, St. Louis, MO, USA) was dissolved in dimethyl sulfoxide (Sigma, St. Louis, MO, USA) and then diluted in normal saline. For the dose-response study, apocynin at 2.5, 5, or 10 mg/kg was intraperitoneally injected immediately and once daily for 10 consecutive days after the operation to determine the optimal dose for the therapy of sepsis-induced cognitive impairments. For the sham groups, the same volume of dimethyl sulfoxide diluted into normal saline was intraperitoneally injected.

### Behavioral and cognitive tests

All behavioral tests were performed at 2:00–5:00 p.m. in a sound-isolated room by the instrument of XR-XZ301 (Xinruan Corporation, Shanghai Soft maze Information Technology Co. Ltd., China). All behavioral data were recorded by the same investigator who was blinded to the animal grouping as described in our previous studies [19, 20].

#### Open field test

The mice were gently placed in the center of a white plastic chamber (40 cm × 40 cm × 40 cm) for 5 min while exploratory behaviors were automatically recorded by a video tracking system. The total distance traveled and the time spent in the center of the open field were recorded. At the end of each test, the surface of the arena was cleaned thoroughly with 75 % alcohol to avoid the presence of olfactory cues.

#### Fear conditioning test

To measure the associative memory, we employed the fear conditioning paradigm (26-cm long × 18-cm wide × 22-cm high). Briefly, the training consisted of a single exposure to a novel context for 3 min, followed by a tone for 30 s (65 dB, 3 kHz) and a single electric foot shock (3 s, 0.75 mA). The contextual fear conditioning test was performed 24 h later by placing the mice back to the same chamber for 5 min. Two hours after the contextual fear conditioning test, the mice were placed into a chamber that was different in shape, color, and smell from the previous one. The training tone was delivered for 3 min to evaluate the tone fear conditioning. The freezing behavior was defined as the absence of all visible movement of the body except for respiration.

### Western blotting analysis

The mice were killed by decapitation, and the brains were removed for determination of the PV, gp91^phox^, and 4-HNE levels at the indicated time points. Briefly, the brain tissues were homogenized on ice using immunoprecipitation buffer (10 mM Tris-HCl, pH 7.4, 150 mM NaCl, 2 mM EDTA, and 0.5 % Nonidet P-40) plus protease inhibitors (1 μg/ml aprotinin, 1 μg/ml leupeptin, and 1 μg/ml pepstatin A). The lysates were collected, centrifuged at 10,000×*g* at 4 °C for 10 min. The supernatant was removed, and protein concentration was determined using the Pierce Bicinchoninic Acid Protein Assay kit (Pierce Technology Co., Iselin, NJ, USA) with bovine serum albumin (BSA) as the standard. Equal amounts of protein were electrophoretically separated on 4–12 % NuPAGE Novex Bis-Tris gradient gels (Invitrogen, NY, USA) and transferred to the polyvinylidene fluoride membranes. After blocking with 5 % non-fat milk for 1 h at room temperature, the membranes were incubated with rabbit anti-PV (1:1000; Abcam, Cambridge, UK), mouse anti-4-HNE (1:500, Abcam), goat anti-gp91^phox^ (1:500; Santa Cruz, Dallas, TX), and mouse anti-GAPDH (1:5000; millipore) overnight at 4 °C, followed by horseradish peroxidase-conjugated secondary antibodies (GE Healthcare, Pittsburgh, PA, USA) for 2 h at room temperature. The protein bands were detected by enhanced chemiluminescence and the quantitation of bands was undertaken using the Image J software (NIH Image, Bethesda, MD, USA).

### Enzyme-linked immunosorbent assay

The mice were killed by decapitation, and the brain tissues were isolated and washed with ice-cold physiological saline to remove the surface blood. The PFC and hippocampus were then separated, weighed, and placed in a homogenizer. The tissue was homogenized with 1-ml ice-cold physiological saline per 100-mg brain tissue. Hypothermal centrifugation was performed at 10,000×*g* for 10 min, and the supernatant was obtained. Standard curves for all cytokines (in duplicates) were generated using the reference cytokine concentrations supplied. Tumor necrosis factor alpha (TNF-α), interleukin (IL)-1β, IL-6, and IL-10 were quantified using specific enzyme-linked immunosorbent assay (ELISA) kits for rats according to the manufacturers’ instructions (R&D Systems, Minneapolis, MN, USA).

### Measurement of malondialdehyde and superoxide dismutase (SOD)

The level of malondialdehyde (MDA) in the PFC and hippocampus, a measure of lipid peroxidation, was assayed in the form of thiobarbituric acid-reactive substances (Jiancheng Bioengineering Institute, Nanjing, China) as described in our previous study [21]. The superoxide dismutase (SOD) activity in the PFC and hippocampus was determined using a SOD assay kit (Jiancheng Bioengineering Institute, Nanjing, China) as we previously described [21]. The MDA level is expressed as nmol/mg protein, and the SOD activity is expressed as U/mg protein.

### Immunofluorescence

The mice were deeply anesthetized with 2 % sodium pentobarbital in saline (60 mg/kg, intraperitoneally; Sigma Chemical Co., St. Louis, MO) and transcardially perfused with physiological saline, followed by 4 % paraformaldehyde in phosphate-buffered saline (PBS; pH = 7.4). The brains were immediately removed, postfixed in the same 4 % paraformaldehyde for 2 h, and dehydrated in 30 % sucrose at 4 °C overnight. They were embedded in optimum cutting compound and cut into 10-μm-thick coronal sections on freezing microtome. The brain sections were mounted on glass slides. The sections were blocked with 1 % BSA for 1 h at room temperature and followed by incubating them with the following primary antibodies: rabbit anti-PV (1:600; Abcam), mouse anti-PV (1:600; Millipore), goat anti-gp91^phox^ (1:200; Santa Cruz), and mouse anti-8-OH-dG (1:200; Santa Cruz) in 1 % BSA at 4 °C overnight. After three washes with PBS, the sections were incubated with the secondary antibodies, goat anti-rabbit IgG-flurescein isothiocyanate (FITC), or Cy3 (1:300; Santa Cruz), goat anti-mouse IgG-FITC or Cy3 (1:600; Bioworld, San Diego, USA), and donkey anti-goat IgG-Cy3 (1:800; Abcam) for 1 h at room temperature. After washing out the secondary antibody, the sections were incubated with 4′,6-diamidino-2-phenylindole for nuclear staining. Fluorescent images were captured by a confocal microscope (Olympus, Japan).

### Neuronal cultures and immunostaining

Primary hippocampal neuronal cultures were prepared according to a previously described protocol with minor modifications [22, 23]. The brains were removed quickly from C57BL/6 mice within 24 h after birth, and the hippocampus was dissected and mechanically disaggregated by gentle trituration using a Pasteur pipette. Dissociated cells were cultured in the neurobasal medium supplemented with 50 × B27, 100 × L-glutamine (200 mM), and 100 × penicillin/streptomycin. The cells were plated at a density of ~1.4 × 10^4^ cells per cm^2^ in polylysine-coated 15-mm multi-well plates. Every 3 days, half of the medium was replaced by freshly prepared medium. Fourteen days after culturing, apocynin (0.5 mM) was added alone or in combination with lipopolysaccharide (LPS) (1 μg/ml). Twenty-four hours later, the neurons in culture were fixed.

For the immunostaining, dissociated neurons were fixed for 10 min at room temperature with 4 % paraformaldehyde/4 % sucrose mixture in PBS that was warmed to 37 °C in advance. The neurons were then permeabilized by 0.1 % Triton X-100 solution in PBS for 10 min at room temperature and washed gently. The neurons were blocked by 1 % BSA in PBS for 1 h at room temperature and incubated with the following primary antibodies: rabbit anti-PV (1:600; Abcam), goat anti-gp91^phox^ (1:200; Santa Cruz), and mouse anti-PSD95 (1:200; Millipore) diluted in 1 % BSA at 4 °C overnight. After three washes with PBS, the neurons were incubated with the secondary antibodies, goat anti-rabbit IgG-FITC (1:300; Santa Cruz), goat anti-mouse IgG-Cy3 (1:600; Bioworld), and donkey anti-goat IgG-Cy3 (1:800; Abcam) for 1 h at room temperature. After washing out the secondary antibody, the neurons were incubated with 4′,6-diamidino-2-phenylindole for nuclear staining. Fluorescent images were obtained by confocal scanning microscopy (Leica, TCS SP2, Germany). For a given sample, 5–10 images, taken at 1 μm intervals, were collapsed to generate a projected image. The PSD-95 puncta number was measured by the Image J (NIH).

### Statistical analysis

Statistical analyses were performed by the Statistical Product for Social Sciences (SPSS; version 16.0, IL, USA). Data are presented as mean ± SEM. Distribution of data was analyzed using the Kolmogorov-Smirnov test. Differences among means were assessed by one-way analysis of variance (ANOVA) followed by a Tukey test. The survival rate was plotted by the Kaplan-Meier method and compared by the log-rank test. Bivariate relationship was evaluated by the Pearson correlation coefficients. A *p* < 0.05 was regarded as statistically significant.

## Results

### Dose-response of apocynin on fear conditioning test and effects of apocynin treatment on the survival rate of sepsis mice

As indicated in Fig. 1b, the dose-response study of apocynin showed that 5 mg/kg was the optimal dose effective in increasing the freezing behavior. Thus, an apocynin dose of 5 mg/kg was used in our subsequent experiments. In addition, apocynin treatment did not affect the survival rate of sepsis mice (Fig. 1c).

### Sepsis-induced cognitive impairments were rescued by apocynin treatment

**Fig. 2:**
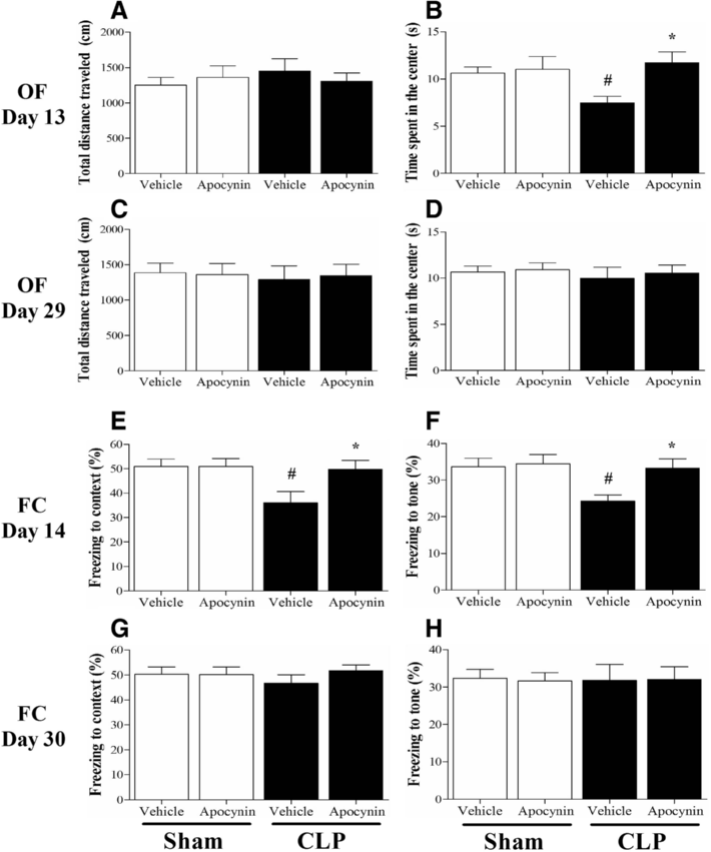
Sepsis-induced cognitive impairments were rescued by apocynin treatment. **a**, **c** No significant difference was observed in the total distance traveled in the open field test at days 13 and 29 among the four groups. **b**, **d** The time spent in the center of the arena was significantly decreased at day 13 but not at day 29 in the CLP + vehicle group compared with the sham groups, whereas apocynin treatment reversed the decrease in the CLP mice at day 13. **e**, **f** The freezing time to context and the freezing time to tone were significantly decreased in the CLP + vehicle group compared with the sham groups at day 14, whereas apocynin treatment reversed the decrease in the CLP mice. **g**, **h** No significant difference was observed in the freezing time to context or the freezing time to tone at day 30 among the four groups. Data are presented as mean ± SEM (*n* = 9–11). ^#^ *p* < 0.05 vs the sham groups, *p < 0.05 vs the CLP + vehicle group

As revealed in Fig. 2a, c, no significant difference was observed in the total distance traveled in the open field test at days 13 and 29 among the four groups [one-way ANOVA; day 13: *F* (3, 36) = 0.839, *p* = 0.678; day 29: *F* (3, 36) = 0.364, *p* = 0.784], suggesting sepsis did not affect the general locomotor activity. However, we observed that sepsis induced significantly less time spent in the center of the arena at day 13 [one-way ANOVA; day 13: *F* (3, 36) = 6.009, *p* = 0.004; Fig. 2b] but not at day 29 compared with the sham groups, indicating sepsis induced the anxiety behavior. Although sepsis-induced cognitive impairments recovered at day 30, the freezing time to context and the freezing time to tone were significantly decreased in the CLP + vehicle group than those in the sham groups at day 14, whereas apocynin treatment reversed the decrease in the CLP mice [one-way ANOVA; context: *F* (3, 36) = 4.533, *p* = 0.009; cue: *F* (3, 36) = 3.926, *p* = 0.016; Fig. 2e, f].

### Sepsis-induced inflammation and oxidative stress in the brain were rescued by apocynin treatment

**Fig. 3:**
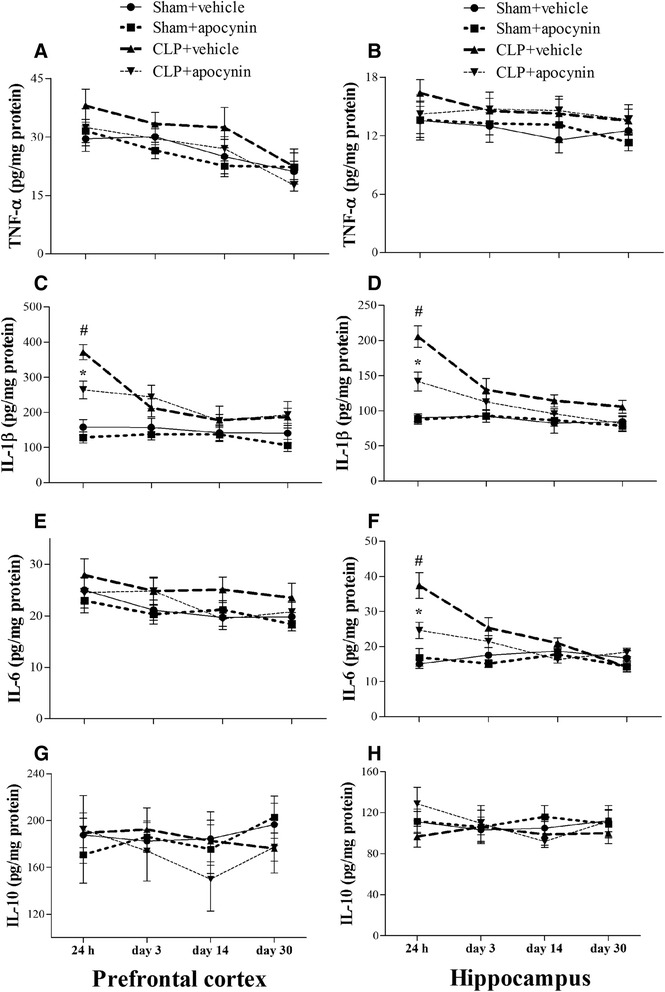
Sepsis-induced neuroninflammation was attenuated by apocynin treatment. **a**, **b** No significant difference was observed in the TNF-α level in the PFC and hippocampus among the four groups over time. **c**, **d** The IL-1β level was significantly increased in the PFC and hippocampus (24 h) in the CLP + vehicle group compared with the sham groups. Apocynin treatment significantly decreased the IL-1β level in the PFC and hippocampus (24 h) in the CLP + apocynin group compared with the CLP + vehicle group. There was no significant difference at other time points among the four groups. **e**, **f** The IL-6 level was significantly increased in the hippocampus but not in the PFC (24 h) in the CLP + vehicle group compared with the sham groups. Apocynin treatment significantly decreased the IL-6 level in the hippocampus (24 h) in the CLP + apocynin group compared with the CLP + vehicle group. There was no significant difference at other time points among the four groups. **g**, **h** No significant difference was observed in the IL-10 level in the PFC and hippocampus among the four groups. Data are presented as mean ± SEM (*n* = 6). ^#^ *p* < 0.05 vs the sham groups, *p < 0.05 vs the CLP + vehicle group

To evaluate the neuroinflammation in the sepsis survivors, we measured TNF-α, IL-1β, IL-6, and IL-10 expressions in the PFC and hippocampus. As shown in Fig. 3, sepsis up-regulated the neuroinflammatory responses as evidenced by the significantly increased pro-inflammatory mediators in the PFC (IL-1β) and hippocampus (IL-1β and IL-6) at 24 h when compared with the sham groups, while apocynin treatment attenuated IL-1β and IL-6 expressions at 24 h after sepsis development [one-way ANOVA; PFC (IL-1β): *F* (3, 20) = 26.575, *p* = 0.01; hippocampus (IL-1β): *F* (3, 20) = 25.889, *p* = 0.03; hippocampus (IL-6): *F* (3, 20) = 15.26, *p* = 0.012; Fig. 3c, d, f].

**Fig. 4:**
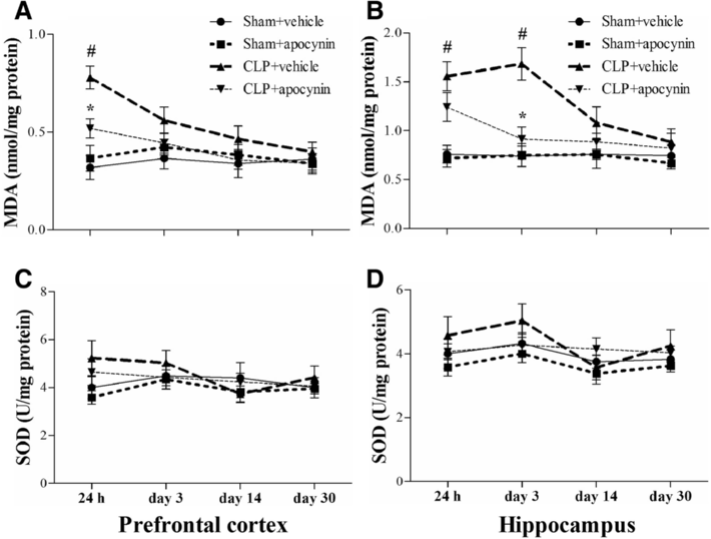
Sepsis-induced oxidative damage was attenuated by apocynin treatment. **a**, **b** The MDA level was significantly increased in the PFC (24 h) and hippocampus (24 h and day 3) in the CLP + vehicle group compared with the sham groups. Apocynin treatment significantly decreased the MDA level in the PFC (24 h) and hippocampus (day 3) in the CLP + apocynin group compared with the CLP + vehicle group. There was no significant difference at other time points among the four groups. **c**, **d** No significant difference was observed in the SOD activity in the PFC and hippocampus among the four groups. Data are presented as mean ± SEM (*n* = 6). ^#^ *p* < 0.05 vs the sham groups; ^*^ *p* < 0.05 vs the CLP + vehicle group

**Fig. 5:**
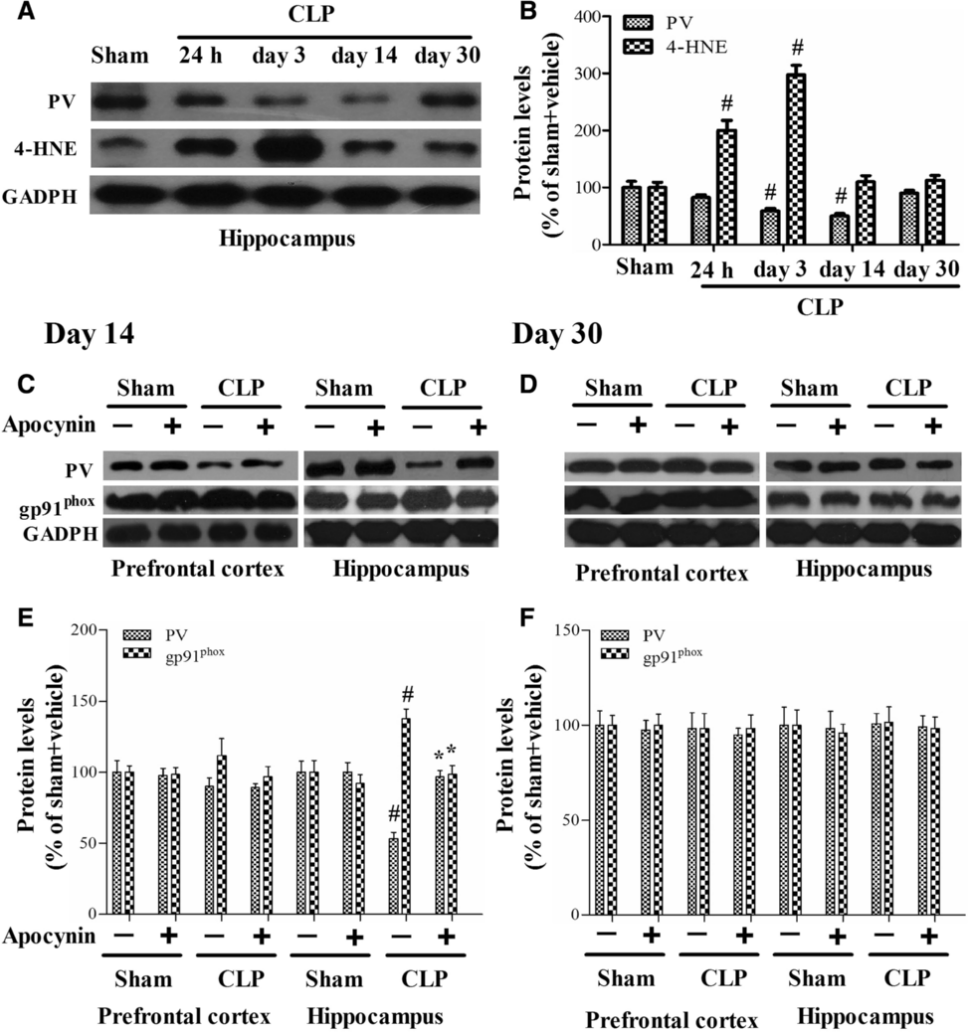
The levels of PV, 4-HNE, and gp91^phox^ in the PFC and hippocampus. **a**, **b** Sepsis induced significant decreases in the PV level from day 3 to day 14 and significant increases in the 4-HNE level from 24 h to day 3 in the hippocampus. **c**–**f** The PV level was decreased and the gp91^phox^ level was increased in the hippocampus at day 14 but not at day 30 in the CLP + vehicle group compared with the sham groups, whereas apocynin treatment reversed these abnormities. There was no significant difference in the PV and gp91^phox^ levels in the PFC among the four groups. Data are presented as mean ± SEM (*n* = 6). ^#^ *p* < 0.05 vs the sham groups; *p < 0.05 vs the CLP + vehicle group

**Fig. 6:**
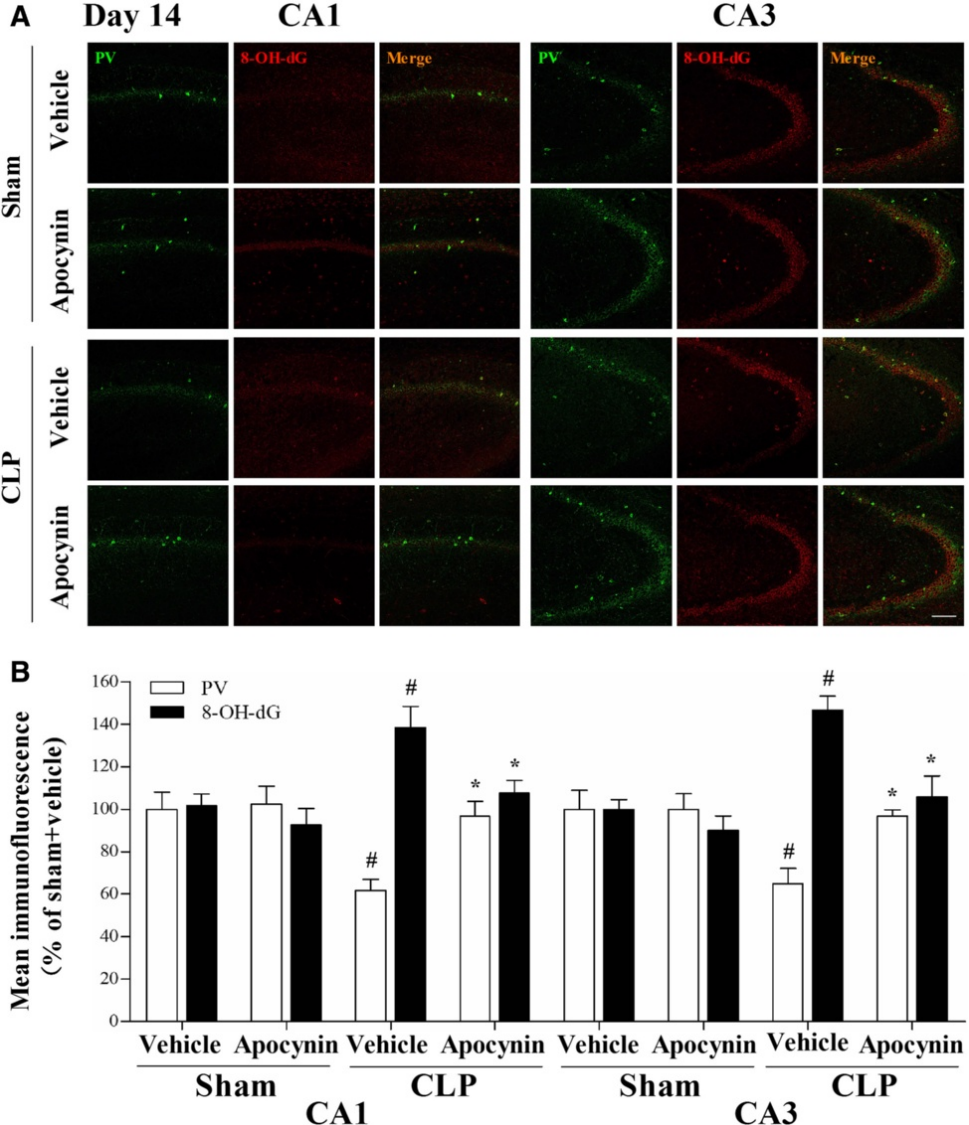
Double-immunofluorescence staining to detect co-localization of PV and 8-OH-dG in the CA1 and CA3 regions of the hippocampus at day 14 after operation. **a** Representative images of PV (*green*) and 8-OH-dG (*red*) in the CA1 and CA3 regions of the hippocampus. **b** The PV immunoreactivity was decreased and the 8-OH-dG immunoreactivity was increased in the PV interneurons in the CA1 and CA3 regions of the hippocampus in the CLP + vehicle group compared with the sham groups, whereas apocynin treatment reversed these abnormities. Data are presented as mean ± SEM (*n* = 6). ^#^ *p* < 0.05 vs the sham groups; *p < 0.05 vs the CLP + vehicle group. *Scale bar* = 100 μm

**Fig. 7:**
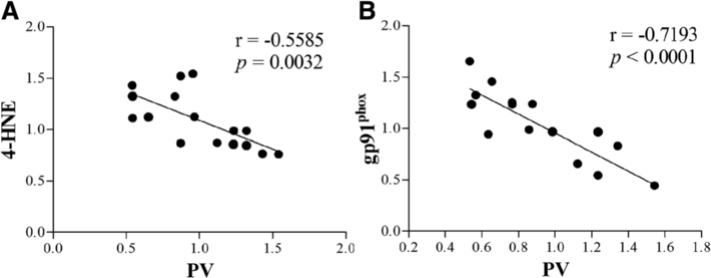
**a**, **b** The PV levels were negatively correlated with the 4-HNE and gp91^phox^ levels

To assess oxidative damage in the brain of the septic mice, we measured MDA and 4-HNE levels, two markers of lipid peroxidation. We observed that MDA level was significantly increased in the PFC (24 h) and hippocampus (24 h and day 3) in the CLP + vehicle group, whereas apocynin treatment attenuated MDA level after sepsis development [one-way ANOVA; PFC (24 h): *F* (3, 20) = 12.437, *p* = 0.025; hippocampus (24 h): *F* (3, 20) = 10.953, *p* = 0.03; hippocampus (day 3): *F* (3, 20) = 11.982, *p* = 0.002; Fig. 4a, b]. In addition, we observed a progressive increase in 4-HNE level in the hippocampus within the first 3 days in the CLP + vehicle group when compared with the sham group (*p* < 0.05; Fig. 5a, b). Furthermore, the increased 8-OH-dG expression in the PV interneurons was prevented by apocynin treatment at day 14 after CLP [PV: one-way ANOVA; CA1: *F* (3, 20) = 6.838, *p* = 0.002; CA3: *F* (3, 20) = 6.009, *p* = 0.004]; [8-OH-dG: one-way ANOVA; CA1: *F* (3, 20) = 6.778, *p* = 0.002; CA3: *F* (3, 20) = 11.898, *p* < 0.01; Fig. 6]. However, no difference in anti-inflammatory IL-10 (*p* > 0.05; Fig. 3g, h) or antioxidant enzyme SOD was observed among the four groups over time (*p* > 0.05; Fig. 4c, d). Linear regression analysis showed that PV levels were negatively correlated with the levels of 4-HNE (*r* = −0.5585, *p* = 0.0032; Fig. 7a).

### Sepsis-induced selective PV interneuron phenotype loss was associated with gp91^phox^ activation

Sepsis induced a progressive decrease in PV level from day 3 to day 14. PV level was significantly decreased while gp91^phox^ expression was increased in the hippocampus at day 14 but not day 30 in the CLP + vehicle group when compared with the sham groups. However, apocynin treatment reversed these abnormities after sepsis development [14 days: one-way ANOVA; PFC (PV): *F* (3, 20) = 0.873, *P* = 0.471; hippocampus (PV): *F* (3, 20) = 14.661, *P* < 0.01]; [14 days: one-way ANOVA; PFC (gp91^phox^): *F* (3, 20) = 0.782, *P* = 0.518; hippocampus (gp91^phox^): *F* (3, 20) = 5.585, *P* = 0.01; Fig. 5c, e]; [30 days: one-way ANOVA; PFC (PV): *F* (3, 20) = 0.104, *P* = 0.957; hippocampus (PV): *F* (3, 20) = 0.019, *P* = 0.996]; [30 days: one-way ANOVA; PFC (gp91^phox^): *F* (3, 20) = 0.022, *P* = 0.996; hippocampus (gp91^phox^): *F* (3, 20) = 0.124, *P* = 0.945; Fig. 5d, f]. Furthermore, linear regression analysis showed that PV levels were negatively correlated with the expressions of gp91^phox^ (*r* = −0.7193, *p* < 0.0001; Fig. 7b).

**Fig. 8:**
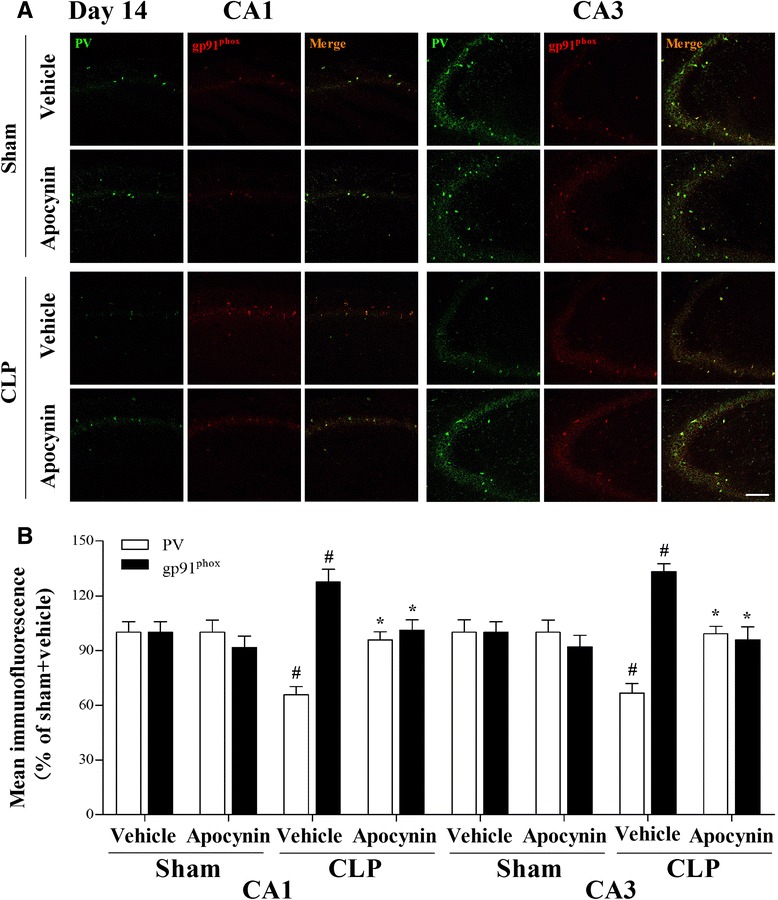
Double-immunofluorescence staining to detect co-localization of PV and gp91^phox^ in the CA1 and CA3 regions of the hippocampus at day 14 after operation. **a** Representative images of PV (*green*) and gp91^phox^ (*red*) in the CA1 and CA3 regions of the hippocampus. **b** The PV immunoreactivity was decreased and the gp91^phox^ immunoreactivity was increased in the PV interneurons in the CA1 and CA3 regions of the hippocampus in the CLP + vehicle group compared with the sham groups, whereas apocynin treatment reversed these abnormities. Data are presented as mean ± SEM (*n* = 6). ^#^ *p* < 0.05 vs the sham groups; *p < 0.05 vs the CLP + vehicle group. *Scale bar* = 100 μm

**Fig. 9:**
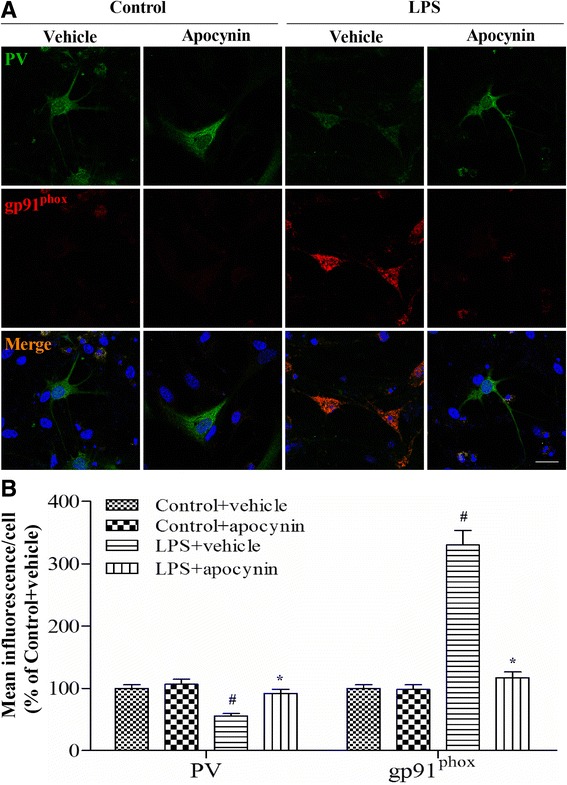
Double-immunofluorescence staining to detect co-localization of PV and gp91^phox^ in the primary hippocampal neuronal cultures. **a** Representative images of PV (*green*) and gp91^phox^ (*red*) in the primary hippocampal neuronal cultures. **b** The PV immunoreactivity was decreased and the gp91^phox^ immunoreactivity was increased in the primary neuronal cultures in the LPS + vehicle group compared with the control groups, whereas apocynin treatment reversed these abnormities. Data are presented as mean ± SEM (*n* = 6). ^#^ *p* < 0.05 vs the control groups; *p < 0.05 vs the LPS + vehicle group. *Scale bar* = 20 μm

To further test the hypothesis that gp91^phox^ activation in the PV interneurons contributes to PV interneuron phenotype loss, we performed double-immunofluorescent labeling for PV and gp91^phox^. As revealed in Fig. 8, gp91^phox^ immunoreactivity mainly colocalized to PV interneurons and most PV-positive cells were also immunoreactive for gp91^phox^. The PV immunoreactivity decreased while gp91^phox^ immunoreactivity increased in the hippocampus at day 14 but not at day 30 (data not shown) in the CLP + vehicle group when compared with the sham groups. However, apocynin treatment reversed these abnormities after sepsis development [one-way ANOVA; CA1 (PV): *F* (3, 20) = 9.397, *p* < 0.01; CA3: *F* (3, 20) = 8.753, *p* < 0.01; CA1 (gp91^phox^): *F* (3, 20) = 6.227, *p* = 0.004; CA3 (gp91^phox^): *F* (3, 20) = 10.183, *p* < 0.01]. There was no difference in PV or gp91^phox^ expression at either day 14 or day 30 in the PV interneurons between groups (data not shown). Supporting those in vivo data, the in vitro study showed that apocynin was able to prevent the increase in gp91^phox^ and the decrease in PV level after LPS stimulation [one-way ANOVA; gp91^phox^: *F* (3, 20) = 11.74, *p* < 0.01; PV: *F* (3, 20) = 13.85, *p* < 0.01; Fig. 9a, b].

### Sepsis resulted in decreased post-synaptic density protein 95 (PSD-95) puncta numbers in the PV interneurons

**Fig. 10:**
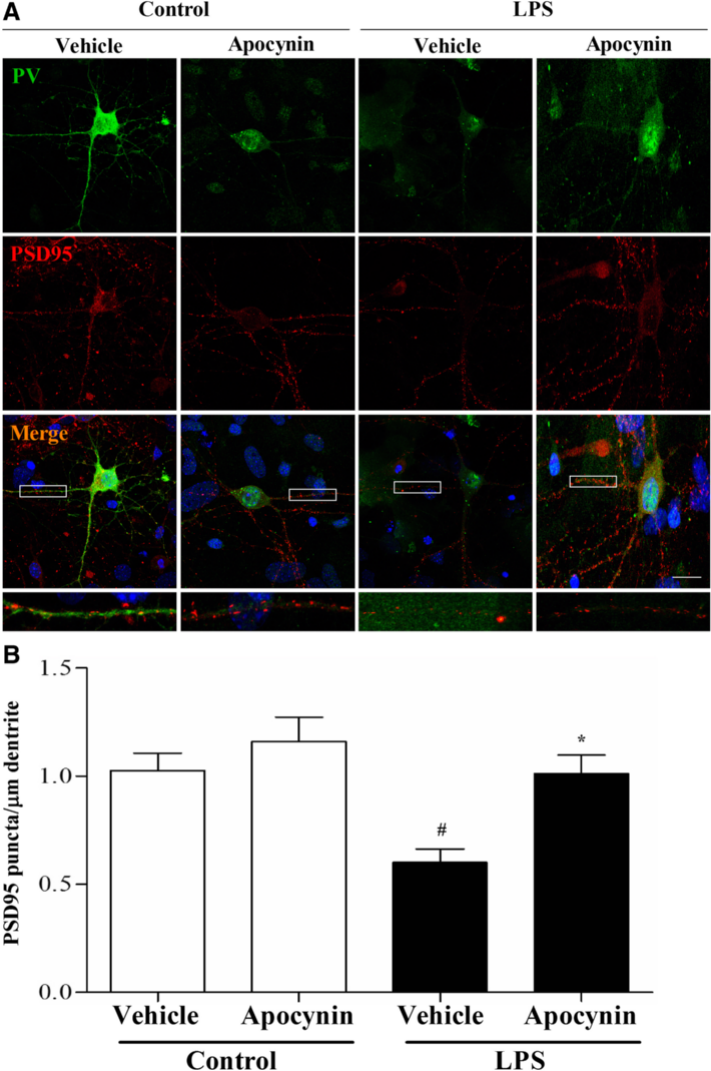
LPS exposure decreased the number of excitatory synapses in the PV interneurons. **a** Representative images of PV (*green*) and PSD-95 (*red*) in the primary hippocampal neuronal cultures. **b** Apocynin treatment reversed LPS exposure-induced decrease in the number of PSD95 puncta in the PV interneurons. Data are presented as mean ± SEM (*n* = 6). ^#^ *p* < 0.05 vs the control groups; *p < 0.05 vs the LPS + vehicle group. *Scale bar* = 20 μm

To investigate the mechanisms underlying sepsis-induced PV interneuron dysfunction, we examined PSD-95, a major scaffolding protein located at excitatory synapses. The in vitro study revealed that LPS led to significantly fewer PSD-95 puncta numbers when compared with the LPS + vehicle group. Notably, apocynin treatment significantly increased PSD-95 puncta number after LPS exposure [one-way ANOVA; *F* (3, 28) = 7.863, *p* = 0.01; Fig. 10a, b].

## Discussion

Our results confirmed previous finding that sepsis induced gradual behavioral deficits during follow-up [24], suggesting CLP-induced cognitive impairments are time dependent. More importantly, we demonstrated that sepsis-induced cognitive impairments co-occurred with hippocampal PV interneuron phenotype loss. However, chronic treatment with apocynin, a NADPH inhibitor and reactive oxygen species (ROS) scavenger, rescued these abnormities. These results demonstrated a critical role of PV interneurons in sepsis-induced brain dysfunction.

The pathogenesis of sepsis-induced cognitive impairments is not completely understood. Recent studies implicate the involvement of oxidative stress and neuroinflammation [5]. An extensive body of evidence indicates that sepsis is associated with increased ROS levels, depletion of antioxidants, and accumulation of markers of oxidative stress, which is intimately linked to a variety of pathophysiological processes, such as enhanced inflammatory responses, mitochondrial dysfunction, and hypoactive N-methyl-D-aspartate receptors [5, 18]. Our results also showed that brain oxidative damage and inflammation were critically involved in the development of cognitive impairments after sepsis development, whereas treatment with apocynin abolished the increases in brain oxidative stress and inflammation markers, and ultimately attenuated the cognitive impairments in the sepsis survivors. However, the mechanisms by which these pathological events lead to sepsis-induced cognitive impairments remain unclear.

PV interneurons comprise about 40–50 % of the total GABAergic population, which regulate the activity of neural networks through perisomatic inhibition onto the pyramidal cells [6]. Synchronous activity of the PV interneurons generates gamma oscillations (30–80 Hz), which is important for cognition, learning, and memory [9]. By contrast, PV interneuron abnormities have been proposed to play a causal role in the development of cognition impairments associated with major psychiatric disorders [6]. Here, we reported hippocampal PV interneuron phenotype loss co-occurred with the cognitive impairments during sepsis development. This selective loss of PV interneuron phenotype might be explained by the notion that the hippocampus is vulnerable to inflammation than the PFC. Notably, these abnormalities were precluded in the mice treated with apocynin, providing evidence that PV interneuron phenotype loss and cognitive impairments may be causally linked.

Although the cause of PV interneuron dysfunction in sepsis is not yet understood, PV interneurons have been proposed to be vulnerable to oxidative stress [15–17]. Mitochondria is considered to be the major sources of intracellular ROS, but accumulating evidence suggests that various subunits of NADPH oxidase in the plasma membrane are highly expressed in cortical neurons and can also generate ROS [25]. Activation of NADPH oxidase leads to the generation of the superoxide ion, a ROS that can be converted to the highly reactive hydroxyl radical and to peroxynitrite, a highly damaging reactive nitrogen species [25, 26]. The NADPH oxidase complex is composed of two membrane-bound subunits (gp91^phox^ and p22^phox^) and four cytoplasmic subunits (p40^phox^, p47^phox^, p67^phox^, and the small G-protein Rac) [27, 28]. Specifically, Nox2-derived ROS production plays a critical role in GABAergic interneuron dysfunction after subchronic ketamine exposure in mice [17]. In the present study, treating animals with apocynin reduced the oxidative stress and prevented the loss of PV immunoreactivity induced by sepsis. Because of the tight link between oxidative stress and inflammation, it is not surprising that pro-inflammatory molecules affect the function of PV interneurons. This notion is supported by the findings that IL-6 mediates ketamine-induced up-regulation of Nox2 and subsequent deficits of PV interneurons [23], while a nonsteroidal anti-inflammatory drug treatment prevents PV interneuron dysfunction following maternal separation [29].

To further test the hypothesis that Nox2 activation specifically in the PV interneurons contributes to oxidative stress and subsequent PV interneuron phenotype loss, we performed double-immunofluorescent labeling for PV and gp91^phox^ immunoreactivity. Our data showed that Nox2 was mainly expressed in the PV interneurons, and there was an inverse relationship between PV and Nox2 expressions, suggesting that reduced PV level is associated with elevated oxidative stress in the PV interneurons. Our results are supported by previous study demonstrating that Nox2 up-regulation appears to be responsible for the prolonged oxidative insult on GABAergic neurons [16]. On the other hand, it has been reported that Nox2 is required for glial cell activation and emphasizes the critical role of oxidative damage and Nox2-derived ROS as central factors contributing to sepsis-induced cognitive impairments [25]. Here, we provide additional evidence that Nox2 activation specifically in the PV interneurons at least in part plays an important role in sepsis-induced PV interneuron phenotype loss and subsequent cognitive impairments. However, our data did not exclude the contribution of ROS from other regions such as mitochondria.

The cerebral cortex is comprised of both excitatory (glutamatergic) neurons and inhibitory (GABAergic) interneurons that work together to maintain the balance between inhibition and excitation that is necessary for the normal functioning of the cortex [30]. The excitability of PV interneurons relies on the summation of excitatory and inhibitory signals, which in turn is regulated by the number of excitatory and inhibitory synapses on them [22]. It has been suggested that disrupted excitatory synapse maturation in the GABAergic interneurons is associated with neuropsychiatric disorders such as schizophrenia [31]. To investigate whether the activity of PV interneurons is changed after sepsis development, we examined PSD-95 and revealed that sepsis significantly reduced the number of PSD-95 puncta in the PV interneurons. Intriguingly, apocynin treatment was able to rescue the decreased PSD-95 puncta number. These findings suggest that maintaining the balance between excitation and inhibition is important for the homeostatic control of normal brain function and behaviors.

There are few limitations in our study protocol. For example, we only performed the open field and fear conditioning tests to reflect some of the behavioral alternations after sepsis development. Therefore, future studies are needed to assess other behavioral changes caused by sepsis, including the spatial working memory. Secondly, sepsis induced the amygdala-dependent memory impairments in the present study, thus the role of PV interneurons in the amygdala deserves great research interests.

In conclusion, our study demonstrated that Nox2 activation, at least in part, plays a key role in sepsis-induced PV interneuron phenotype loss and subsequent cognitive impairments. Hence, identifying viable therapeutic strategies to tackle on oxidative stress and neuroinflammation and the consequent PV interneuron disturbances may provide a therapeutic strategy for SAE.
